# Personality beyond the brain: a new perspective on the brain-gut-gonadal axis and human fertility

**DOI:** 10.3389/fnbeh.2026.1985122

**Published:** 2026-10-13

**Authors:** Bogdan Doroftei, Ahmed Adel Mansour Kamar, Alin Ciobica, Dragos Puia, Mara Doroftei, David Puha, Bogdan Puha

**Affiliations:** 1 Origyn Fertility Center, Iași, Romania; 2 Faculty of Medicine, University of Medicine and Pharmacy, “Grigore T. Popa”, Iasi, Romania; 3 Spitalul Clinic de Obstetrică și Ginecologie “Cuza Vodă”, Iasi, Romania; 4 Doctoral School of Biology, Faculty of Biology, Alexandru Ioan Cuza University of Iași, Iasi, Romania; 5 Department Biology, Faculty of Biology, IASI, Alexandru Ioan Cuza University (UAIC), City Iasi, Romania; 6 Department of Urology, “Dr. C.I. Parhon” Clinical Hospital, Iasi, Romania; 7 Sf. Spiridon Emergency Clinical Hospital of Iasi, Iasi, Romania; 8 Department of Orthopedics and Traumatology, “Grigore T. Popa” University of Medicine and Pharmacy, Iasi, Romania

**Keywords:** autonomic nervous system, brain–gut axis, infertility, neuroendocrine regulation, personality traits, physiological personality phenotype, reproductive health, sexual dysfunction

## Abstract

Despite major advances in reproductive medicine, many cases of infertility remain unexplained. Hormonal, genetic, anatomical, and environmental factors have been extensively investigated, whereas the possible contribution of personality characteristics to reproductive physiology has received little attention. Throughout this review, personality refers to relatively consistent individual characteristics and should not be confused with temporary emotional states or acute psychological stress. Published evidence reports associations between personality characteristics and measures of autonomic and neuroendocrine regulation, gastrointestinal symptoms or function, sexual function, and reproductive health. However, the strength and type of evidence vary across these domains, and most findings are observational or associative rather than causal. Established biological pathways should therefore be distinguished from reported personality-related associations and from the hypothetical cross-system links proposed in this review. This Mini Review examines current evidence linking personality characteristics with autonomic and neuroendocrine regulation, the brain–gut axis, the hypothalamic–pituitary–gonadal axis, sexual function, and fertility. Rather than proposing that internal organs possess personalities, we propose that shared neurobiological and neuroendocrine mechanisms may produce parallel functional response patterns that reflect an individual’s personality characteristics. Finally, we propose that integrating these previously studied relationships within a Brain–Gut–Gonadal framework may provide a new perspective on the possible links between personality traits, sexual function, reproductive physiology, and unexplained infertility. This perspective does not imply that personality traits cause infertility, but raises the novel and testable possibility that personality-related biological differences may contribute to individual variation in reproductive physiology and fertility.

## 1 Introduction

Infertility is a major global health problem affecting millions of couples worldwide. Although considerable progress has been achieved in understanding the genetic, hormonal, anatomical, environmental, and lifestyle determinants of infertility, a substantial proportion of cases remain unexplained. This suggests that additional biological factors contributing to reproductive physiology have not yet been fully recognized (Qiu et al., 2023; Li et al., 2024; Lv et al., 2024).

Personality characteristics are relatively stable individual patterns of thinking, feeling, and behavior that are increasingly recognized as having identifiable neurobiological foundations rather than representing purely psychological phenomena (DeYoung, 2010). Throughout this review, the term *personality* refers to these relatively consistent individual characteristics and should not be confused with temporary emotional states, acute stress, or transient mood changes.

Growing evidence indicates that personality traits are associated with differences in physiological regulation beyond behavior, including variation in hypothalamic–pituitary–adrenal (HPA) axis reactivity (Montoliu et al., 2020). Associations have also been reported between psychological or personality-related traits and autonomic regulation (Nardelli et al., 2015), inflammatory activity (Wagner et al., 2019), and dermatological conditions (Lugović-Mihić et al., 2021), alongside the gastrointestinal and reproductive associations discussed below. These findings represent different levels of evidence. Reported associations between personality characteristics and physiological measures are empirical observations, whereas the mechanisms that may explain these associations remain incompletely established. Their integration within the proposed Brain–Gut–Gonadal framework is therefore hypothesis-generating and should not be interpreted as evidence that personality directly regulates gastrointestinal or reproductive physiology.

Studies have reported relationships between personality characteristics and sexual behavior, sexual satisfaction, sexual dysfunction, and reproductive health (Cabello-García et al., 2024; Mencarini et al., 2022; Darolia and Ghosh, 2022; Allen, 2019; Burgio et al., 2024; Chang et al., 2025; Grevenstein and Bluemke, 2026; Dragan et al., 2020). Similarly, personality traits have been associated with functional gastrointestinal disorders, bowel symptoms, and irritable bowel syndrome, supporting the concept that gastrointestinal physiology may vary according to individual personality characteristics (Cryan et al., 2022; Rangraze, 2025; Guo et al., 2022; Groen et al., 2025; Muscatello et al., 2016; Tayama et al., 2012). In parallel, increasing evidence highlights the importance of the brain–gut axis and the gut microbiota in reproductive physiology and fertility in both males and females (Li et al., 2024; Lv et al., 2024; Cryan et al., 2022; Moustakli et al., 2025).

Although associations between personality traits and autonomic, endocrine, gastrointestinal, and reproductive processes have been studied independently, their possible relevance to fertility has received considerably less attention (Mencarini et al., 2022; Allen, 2019; Peters, 2023; Dragan et al., 2020). Recent evidence also suggests associations between personality-related traits and female reproductive conditions, although the biological mechanisms and direction of these relationships remain uncertain (Kong et al., 2024). In particular, it remains unclear whether personality-related biological differences may be reflected in coordinated gastrointestinal and reproductive responses through shared neurobiological, autonomic, endocrine, or immune mechanisms.

In this Mini Review, we bring together evidence from personality neuroscience, brain–gut interactions, reproductive physiology, and sexual medicine to examine whether personality-related biological differences may be associated with parallel responses in the gastrointestinal and reproductive systems. We focus particularly on the possible relevance of these relationships to reproductive physiology and unexplained infertility and identify the main biological mechanisms and research questions that require further investigation.

The Brain–Gut–Gonadal perspective therefore does not assume that the individual links between personality and physiological regulation are new. Rather, it brings these findings together to examine a less explored question: whether personality-related biological variability may be relevant to reproductive physiology and unexplained infertility. The proposed conceptual framework and its potential points of interaction are summarized in Figure 1.

**Figure 1:**
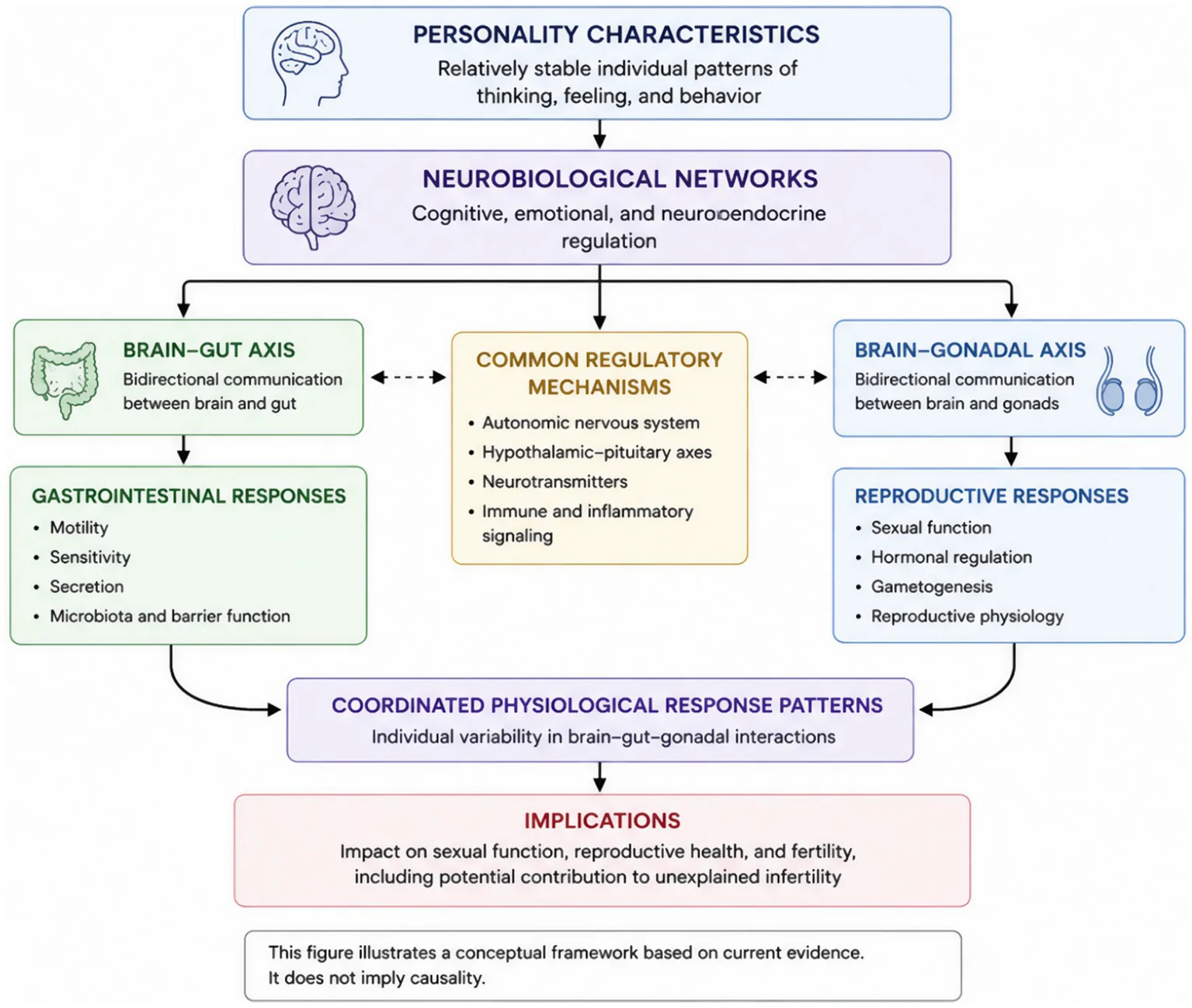
Proposed model of coordinated physiological response patterns associated with personality characteristics. The figure was created by the authors with the assistance of an artificial intelligence (AI) tool for visual design. The scientific content, conceptual framework, and final figure were reviewed and approved by the authors.

The figure represents a conceptual, hypothesis-generating framework rather than an established biological axis. Established physiological pathways should be distinguished from observational associations and proposed cross-system connections. In particular, the proposed links between personality characteristics, gut microbiota, and reproductive physiology remain indirect and require further validation. The framework does not imply causality.

## 2 Methodology and literature search strategy

This Mini Review was conducted to examine the current evidence regarding the relationship between personality characteristics, gastrointestinal physiology, reproductive physiology, sexual function, and fertility. A structured literature search was performed using PubMed/MEDLINE, Scopus, Web of Science, and Google Scholar to identify peer-reviewed articles published in English. The search primarily focused on publications from 2000 to 2026, while earlier landmark studies were included when considered essential for the conceptual background.

The search strategy combined keywords and Medical Subject Headings (MeSH) related to personality traits, temperament, personality disorders, personality neuroscience, brain–gut axis, gut microbiota, autonomic nervous system, hypothalamic–pituitary–adrenal axis, hypothalamic–pituitary–gonadal axis, neuroendocrine regulation, immune and inflammatory pathways, sexual function, reproductive physiology, fertility, infertility, and unexplained infertility. Search terms were combined according to the topic examined and adapted to the syntax of each database. Representative combinations included “personality traits” AND “fertility,” “personality” AND “autonomic nervous system,” “personality” AND “cortisol,” and “gut microbiota” AND “reproductive function”.

Priority was given to systematic reviews, meta-analyses, high-quality narrative reviews, and original studies that directly addressed personality or temperament in relation to neurobiological, autonomic, endocrine, immune, gastrointestinal, sexual, or reproductive outcomes. Studies were also considered when they provided mechanistic evidence regarding HPA–HPG interactions, autonomic regulation, neuroendocrine signaling, and gut–reproductive interactions. Studies focusing exclusively on transient emotional states, acute psychological stress, or mood disorders without relevance to stable personality characteristics were generally excluded. The literature search was last updated in August 2026. Titles and abstracts were screened for eligibility, followed by full-text assessment of potentially relevant articles. The search identified 70 potentially relevant records. After removal of one duplicate, 69 records were screened, of which 32 were excluded because they did not meet the scope or inclusion criteria. Consequently, 37 records were included in the final narrative synthesis.

The evidence was synthesized narratively, with attention to study design, directness to the research question, and the strengths and limitations of the available studies. For interpretation, evidence was considered at three levels: (1) established biological relationships supported by physiological or mechanistic evidence; (2) empirical associations between personality characteristics and autonomic, endocrine, gastrointestinal, sexual, or reproductive outcomes; and (3) hypothetical cross-system connections proposed within the Brain–Gut–Gonadal framework. Associative and hypothetical findings were not interpreted as demonstrating causation. Given the heterogeneity of the included observational, clinical, mechanistic, and review evidence, no meta-analysis or formal risk-of-bias scoring was performed.

## 3 Biological foundations of personality

In this Mini Review, the primary focus is on personality traits as relatively stable individual differences within the general population, rather than on personality disorders. Dimensional models, particularly the Big Five, provide a useful framework for describing such variation (DeYoung, 2010). Temperament is also relevant because developmental models have linked stable individual differences to biological and autonomic reactivity (Ecker et al., 2025; Cloninger et al., 1993). Evidence from personality disorders is considered where it provides additional insight into neurobiological, autonomic, endocrine, or reproductive mechanisms, but personality disorders are not the primary focus of the proposed framework (DeYoung, 2010; Ecker et al., 2025).

Personality characteristics refer to relatively stable patterns of cognition, emotion, and behavior that distinguish individuals across time and contexts. Increasing evidence from personality neuroscience indicates that these traits are not purely descriptive psychological constructs but are rooted in identifiable brain networks involved in emotional regulation, reward processing, and executive control. Rather than being explained by individual neurotransmitter systems or isolated brain regions, personality traits are increasingly understood in relation to distributed and interacting neural networks involved in reward processing, emotional regulation, salience processing, and executive control (DeYoung, 2010).

Within personality neuroscience, personality traits are commonly conceptualized as relatively stable neurobehavioral characteristics rather than transient psychological states. Unlike acute stress, anxiety, or mood fluctuations, personality traits show relative stability across time and have been associated with variation in autonomic, endocrine, immune, and behavioral measures (Montoliu et al., 2020; Wagner et al., 2019). However, these associations do not establish that personality traits directly regulate these physiological systems.

Although this review focuses primarily on normal-range personality traits, evidence from personality disorders is considered where it provides additional insight into relevant neurobiological, autonomic, and neuroendocrine mechanisms (Gutiérrez and Valdesoiro, 2023; Rangraze, 2025). However, personality disorders represent clinically distinct conditions, and findings from these populations should not be directly extrapolated to individuals assessed on normal-range personality dimensions. Such evidence is therefore considered complementary rather than interchangeable with evidence from studies of normal personality traits.

In addition, personality traits have been associated with broader health-related outcomes, including sexual behavior and reproductive health, suggesting systemic biological relevance beyond psychological classification alone (Allen, 2019; Chang et al., 2025). These observations provide the conceptual basis for examining whether personality-related neurobiological traits may be reflected in peripheral physiological systems such as the gastrointestinal and reproductive systems.

## 4 Personality and gastrointestinal physiology

The gastrointestinal system is closely connected to the central nervous system through the brain–gut axis, a bidirectional communication network involving neural, hormonal, and immune signaling pathways (Cryan et al., 2022). This system regulates key digestive functions, including motility, secretion, and visceral sensitivity, and is influenced by higher-order brain processes.

Evidence suggests that personality characteristics may be associated with distinct patterns of gastrointestinal function. Studies on functional gastrointestinal disorders, particularly irritable bowel syndrome (IBS), have shown that personality traits such as neuroticism are associated with altered bowel habits, visceral hypersensitivity, and symptom severity. These findings suggest an association between stable personality traits and gastrointestinal symptoms or functional responses, although they do not establish a direct causal effect of personality on gastrointestinal physiology (Groen et al., 2025; Muscatello et al., 2016; Tayama et al., 2012).

Beyond functional disorders, the gut microbiota has emerged as an important mediator between central nervous system activity and gastrointestinal physiology. Dysbiosis has been associated with altered gut signaling and systemic metabolic, immune, and endocrine changes, some of which may also be relevant to reproductive function (Moustakli et al., 2025; Guo et al., 2022). However, these findings do not establish a direct personality–microbiota–reproductive pathway. The microbiota–gut–brain axis further supports the concept that central behavioral traits and peripheral physiological systems are biologically interconnected (Cryan et al., 2022; Guo et al., 2022). Interpretation of microbiome findings should also consider potential confounding by diet, medication, metabolic status, lifestyle, and other clinical factors.

From a broader perspective, gastrointestinal manifestations associated with personality characteristics may reflect stable autonomic and neuroendocrine response patterns (Montoliu et al., 2020; Nardelli et al., 2015). These patterns may influence gut motility and secretion, potentially explaining why individuals with different personality profiles may present with different functional bowel tendencies.

Together, these observations support an association between personality traits and gastrointestinal function, potentially involving shared autonomic, neuroendocrine, and microbiota-related pathways. However, current evidence does not establish that personality traits directly determine specific gastrointestinal physiological patterns.

## 5 Personality and reproductive physiology

Reproductive physiology is regulated by complex interactions between the hypothalamic–pituitary–gonadal (HPG) axis, the autonomic nervous system, and peripheral endocrine signaling. These systems control sexual development, gonadal function, and reproductive capacity in both males and females (Qiu et al., 2023). Emerging evidence suggests that reproductive function may also be influenced by higher-order behavioral and psychological traits, including personality characteristics. Potential points of convergence include HPA–HPG interactions and autonomic regulation, both of which can influence reproductive endocrine function (Karunyam et al., 2023; Chai et al., 2023; Bendarska-Czerwińska et al., 2023). Autonomic regulation provides one plausible mechanism, as disturbances in autonomic nervous system activity may affect gonadal and reproductive function and have been associated with male infertility (Qiu et al., 2023).

Several studies have reported associations between personality traits and sexual function. In men, personality-related factors have been linked to variations in libido, erectile function, and ejaculatory control, including premature ejaculation (Chang et al., 2025). In women, personality characteristics may influence sexual arousal, satisfaction, and overall sexual response patterns (Grevenstein and Bluemke, 2026). These findings suggest that stable personality traits may be associated with differences in sexual physiological responses rather than only behavioral expression.

In addition, personality traits have been associated with fertility-related and reproductive outcomes (Mencarini et al., 2022; Allen, 2019; Peters, 2023; Kong et al., 2024; Dragan et al., 2020). A systematic review by Darolia and Ghosh (Darolia and Ghosh, 2022), which included 23 studies, identified personality characteristics including neuroticism, harm avoidance, and psychoticism in relation to psychological vulnerability and adjustment to infertility. More recently, Ghosh, Darolia, and Raj (Ghosh et al., 2025) reported significant differences between fertile and infertile couples, with the infertile group showing higher neuroticism and psychoticism, greater sensory sensitivity, and greater interpersonal and sexual conflict. These findings strengthen the evidence for associations between personality-related characteristics and the experience of infertility. However, they do not establish that personality traits cause infertility or directly affect reproductive physiology. Behavioral, psychological, and biological associations should therefore not be considered equivalent. Accordingly, associations with infertility-related psychological adjustment, sexual behavior, fertility outcomes, and directly measured reproductive physiology are treated as distinct forms of evidence in this review.

The direction of these associations also remains uncertain. Pre-existing personality characteristics may influence psychological or behavioral responses to infertility; however, infertility itself and its consequences may also influence personality-related measurements and psychological functioning. Prolonged fertility treatment, sexual difficulties, relationship stress, and uncertainty regarding parenthood may contribute to changes in emotional and behavioral responses. Therefore, observed associations may reflect pre-existing personality characteristics, consequences of infertility, reciprocal relationships, or shared confounding factors rather than a unidirectional personality-to-infertility pathway.

Several biological pathways could provide plausible links between personality-related physiological variation and reproductive function. Personality traits have been associated with differences in HPA-axis activity (Montoliu et al., 2020), while cortisol and HPA-axis dysregulation have been investigated in relation to reproductive function and infertility; findings remain inconsistent (Karunyam et al., 2023; Chai et al., 2023). Interactions between the HPA and HPG axes, together with autonomic and endocrine signaling, therefore represent possible points of convergence rather than established causal pathways (Karunyam et al., 2023; Chai et al., 2023; Bendarska-Czerwińska et al., 2023).

Importantly, these relationships may be bidirectional. Sex steroids can influence brain development and neural function, providing a biological basis for reproductive endocrine activity to affect personality-related neurobehavioral phenotypes rather than personality acting only upstream of reproductive physiology (Garcia-Segura et al., 2023). Neuroimmune mechanisms may provide another point of convergence, as hypothalamic glial cells, including microglia, participate in inflammatory and metabolic signaling that can influence hypothalamic neuroendocrine regulation relevant to reproductive function (Clayton et al., 2022).

Eating behavior may represent an additional point of convergence, as associated changes in metabolic status can influence reproductive physiology, including ovulatory function in females and spermatogenesis in males (Zheng et al., 2024; Crean and Senior, 2021).

The gut microbiota has also been proposed as a contributing factor in reproductive physiology. Dysbiosis has been linked to impaired reproductive function in both males and females, potentially through metabolic, immune, and endocrine pathways (Li et al., 2024; Lv et al., 2024; Moustakli et al., 2025). This further supports the presence of interconnected biological systems linking central regulation, gastrointestinal function, and reproductive health.

Polycystic ovary syndrome (PCOS), also referred to here as polyendocrine metabolic ovarian syndrome (PMOS), provides a relevant clinical example of convergence between reproductive endocrine dysfunction and personality-related characteristics, with differences in personality and temperament reported in affected women (Rzońca et al., 2022).

Overall, current evidence supports several possible points of convergence between personality-related biological variation and reproductive physiology, particularly through autonomic and neuroendocrine pathways. However, direct evidence linking specific personality traits to reproductive physiological mechanisms remains limited, and the proposed relationships should therefore be considered associative and hypothesis-generating rather than causal.

## 6 Discussion and integrated brain–gut–gonadal framework

The evidence presented in this review identifies associations between personality characteristics and several gastrointestinal, sexual, endocrine, and reproductive outcomes. However, direct evidence for coordinated physiological responses across these systems remains limited. Personality characteristics have been associated with variation in autonomic, endocrine, and immune measures, but these empirical associations should be distinguished from the mechanistic pathways that may explain them and from the broader Brain–Gut–Gonadal integration proposed in this review (DeYoung, 2010; Montoliu et al., 2020; Wagner et al., 2019).

The brain–gut axis represents a well-established bidirectional communication system linking central neural processes with gastrointestinal function through neural, endocrine, and immune pathways (Cryan et al., 2022). In parallel, the hypothalamic–pituitary–gonadal (HPG) axis regulates reproductive physiology and sexual function through tightly controlled neuroendocrine signaling (Qiu et al., 2023). Emerging evidence also indicates that gut microbiota may influence both gastrointestinal and reproductive physiology, suggesting additional levels of systemic interaction (Li et al., 2024; Lv et al., 2024; Moustakli et al., 2025).

Within this context, personality-related biological variation may interact bidirectionally with autonomic, neuroendocrine, and reproductive processes rather than functioning solely as an upstream factor (Montoliu et al., 2020; Kong et al., 2024; Karunyam et al., 2023; Chai et al., 2023; Bendarska-Czerwińska et al., 2023). Clinical and observational studies suggest associations between personality traits, gastrointestinal symptoms, and sexual function, although these findings do not demonstrate coordinated physiological responses across these systems (Chang et al., 2025; Grevenstein and Bluemke, 2026; Groen et al., 2025; Muscatello et al., 2016; Tayama et al., 2012). Furthermore, personality traits have been associated with functional gastrointestinal disorders and reproductive outcomes, supporting the presence of cross-system biological associations (Mencarini et al., 2022; Groen et al., 2025; Muscatello et al., 2016; Tayama et al., 2012).

Directionality is particularly important when interpreting studies of personality and infertility. Cross-sectional associations cannot determine whether personality characteristics preceded infertility, developed or changed in response to infertility-related experiences, or reflect reciprocal relationships. Infertility diagnosis, prolonged treatment, sexual difficulties, relationship stress, and uncertainty regarding parenthood may themselves influence psychological functioning and personality-related measurements. In addition, common factors such as age, socioeconomic conditions, lifestyle, comorbidities, and psychological distress may contribute to both personality-related measures and reproductive outcomes.

Evidence from personality disorders may provide additional insight into these relationships, as alterations in emotional regulation and behavioral control have been associated with gastrointestinal symptoms and sexual dysfunction (Gutiérrez and Valdesoiro, 2023; Rangraze, 2025; Cabello-García et al., 2024). Although these relationships remain largely associative, they suggest that personality-related neurobiological differences may be reflected across multiple physiological domains.

Three levels of interpretation should therefore be distinguished. First, established physiological relationships include recognized brain–gut communication and autonomic, neuroendocrine, and immune signaling pathways. Second, observational studies provide empirical associations between personality characteristics and gastrointestinal, sexual, endocrine, or reproductive outcomes. Third, the integration of these observations into a personality-related Brain–Gut–Gonadal framework remains hypothetical and requires direct experimental and longitudinal validation.

The integrated Brain–Gut–Gonadal framework proposed in this Mini Review brings these relationships together as a conceptual, hypothesis-generating model. Established biological pathways, including brain–gut communication and autonomic, neuroendocrine, and immune signaling, provide the physiological basis of the framework (Li et al., 2024; Lv et al., 2024; Cryan et al., 2022; Montoliu et al., 2020; Karunyam et al., 2023; Chai et al., 2023; Bendarska-Czerwińska et al., 2023; Nardelli et al., 2015; Wagner et al., 2019). In contrast, associations between personality characteristics and gastrointestinal or reproductive outcomes are supported mainly by observational evidence, while the proposed links involving personality, gut microbiota, and reproductive physiology remain indirect and require further validation (Moustakli et al., 2025). The framework therefore does not establish a new biological axis or imply causality between its components.

A potentially important application of this framework is unexplained infertility. We do not propose that personality traits are an established cause of infertility or that they currently explain a defined proportion of unexplained infertility. Rather, we propose the testable possibility that personality-related biological variability may contribute to individual differences in reproductive physiology and may therefore warrant investigation in patients with unexplained infertility (Mencarini et al., 2022; Allen, 2019; Peters, 2023; Kong et al., 2024; Karunyam et al., 2023; Chai et al., 2023; Bendarska-Czerwińska et al., 2023; Dragan et al., 2020).

## 7 Future perspectives

The integrated Brain–Gut–Gonadal framework proposed in this review requires systematic validation through well-designed clinical and experimental studies. Future research should investigate whether specific personality traits are associated with measurable physiological patterns across gastrointestinal and reproductive systems using standardized psychometric tools, endocrine profiling, and functional clinical assessments.

Longitudinal cohort studies are needed to determine whether personality traits prospectively predict gastrointestinal, sexual, and reproductive outcomes and to examine the direction of these relationships over time. Studies combining neuroimaging, autonomic assessment, HPA- and HPG-axis measures, inflammatory markers, and reproductive hormones may help identify mechanisms through which these systems converge.

Special attention should be given to unexplained infertility. Studies comparing individuals with unexplained infertility, explained infertility, and fertile controls could examine whether specific personality traits are associated with differences in autonomic activity, cortisol dynamics, reproductive hormones, inflammatory markers, sexual function, or gut microbiota. Such studies would directly test whether personality-related biological variability has relevance to unexplained infertility rather than assuming such a relationship.

Overall, future studies should aim to determine whether personality-related biological variability represents a meaningful factor in reproductive physiology or whether the observed associations reflect parallel but independent physiological processes.

## 8 Limitations

This Mini Review has several limitations. The available evidence is heterogeneous in study design, populations, and methods used to assess personality and physiological outcomes, and most reported relationships are associative. Direct evidence linking personality traits with reproductive physiology remains limited, and the observed associations may be influenced by confounding factors and reverse causality. In addition, the proposed Brain–Gut–Gonadal framework has not yet been validated in longitudinal or experimental studies. It should therefore be considered a conceptual, hypothesis-generating framework rather than an established biological axis.

## 9 Conclusion

This Mini Review brings together evidence linking personality traits with neurobiological, gastrointestinal, sexual, and reproductive processes within a proposed Brain–Gut–Gonadal framework. This model should be regarded as a conceptual, hypothesis-generating framework rather than an established biological axis and highlights possible points of convergence through autonomic, neuroendocrine, immune, and microbiota-related pathways.

Current evidence is heterogeneous and remains predominantly associative. Although established autonomic, neuroendocrine, immune, gastrointestinal, and reproductive pathways provide biological plausibility for cross-system interactions, direct evidence that personality traits influence reproductive physiology or fertility remains limited. Nevertheless, personality-related biological variability may represent an underexplored factor in reproductive physiology and unexplained infertility. Direct mechanistic and longitudinal studies are needed to determine whether these relationships reflect meaningful biological integration or parallel but independent physiological processes.
